# Impact of the great east Japan earthquake on the body mass index of preschool children: a nationwide nursery school survey

**DOI:** 10.1136/bmjopen-2015-010978

**Published:** 2016-04-07

**Authors:** Hiroshi Yokomichi, Wei Zheng, Hiroko Matsubara, Mami Ishikuro, Masahiro Kikuya, Tsuyoshi Isojima, Susumu Yokoya, Toshiaki Tanaka, Noriko Kato, Shoichi Chida, Atsushi Ono, Mitsuaki Hosoya, Soichiro Tanaka, Shinichi Kuriyama, Shigeo Kure, Zentaro Yamagata

**Affiliations:** 1 Department of Health Sciences, University of Yamanashi, Yamanashi, Japan; 2 Department of Social Medicine and Health Education, Peking University, Beijing, China; 3 Department of Disaster Public Health, International Research Institute of Disaster Science, Tohoku University, Miyagi, Japan; 4 Department of Preventive Medicine and Epidemiology, Tohoku Medical Megabank Organization, Miyagi, Japan; 5 Department of Pediatrics, The University of Tokyo, Tokyo, Japan; 6 Center for Clinical Research Data, National Center for Child Health and Development, Tokyo, Japan; 7 Japanese Association for Human Auxology, Tokyo, Japan; 8 Department of Early Childhood Care and Education, Jumonji University, Saitama, Japan; 9 Department of Pediatrics, Iwate Medical University, Iwate, Japan; 10 Department of Pediatrics, Fukushima Medical University, Fukushima, Japan; 11 Department of Pediatrics, Tohoku University, Miyagi, Japan

**Keywords:** body mass index, earthquake, Fukushima nuclear accident, preschool child, growth

## Abstract

**Objective:**

To evaluate the impact of the 2011 great east Japan earthquake on body mass index (BMI) of preschool children.

**Design:**

Retrospective cohort study and ecological study.

**Setting:**

Affected prefectures (Fukushima, Miyagi and Iwate) and unaffected prefectures in northeast Japan.

**Participants:**

The cohort study assessed 2033 and 1707 boys and 1909 and 1658 girls in 3 affected prefectures and unaffected prefectures, respectively, all aged 3–4 years at the time of the earthquake. The ecological study examined random samples of schoolchildren from the affected prefectures.

**Primary and secondary outcome measures:**

The cohort study compared postdisaster changes in BMIs and the prevalence of overweight and obese children. The ecological study evaluated postdisaster changes in the prevalence of overweight children.

**Results:**

1 month after the earthquake, significantly increased BMIs were observed among girls (+0.087 kg/m^2^ vs unaffected prefectures) in Fukushima and among boys and girls (+0.165 and +0.124 kg/m^2^, respectively vs unaffected prefectures) in Iwate. 19 months after the earthquake, significantly increased BMIs were detected among boys and girls (+0.137 and +0.200 kg/m^2^, respectively vs unaffected prefectures) in Fukushima, whereas significantly decreased BMIs were observed among boys and girls (−0.218 and −0.082 kg/m^2^, respectively vs unaffected prefectures) in Miyagi. 1 month after the earthquake, Fukushima, Miyagi and Iwate had a slightly increased prevalence of overweight boys, whereas Fukushima had a slightly decreased prevalence of overweight girls, compared with the unaffected prefectures. The ecological study detected increases in the prevalence of overweight boys and girls in Fukushima who were 6–11 and 6–10 years of age, respectively.

**Conclusions:**

These results suggest that in the affected prefectures, preschool children gained weight immediately after the earthquake. The long-term impact of the earthquake on early childhood growth was more variable among the affected prefectures, possibly as a result of different speeds of recovery.

**Strengths and limitations of this study**

- The study analysed a unique data set on child body mass index before and after a disaster.
- The study establishes a reference group for comparison as children mature.
- The study data were limited to nursery school records.
- The cohort of affected participants did not include those who died or relocated.
- The information on previous diets and physical activities was lacking.

## Introduction

**Figure 1:**
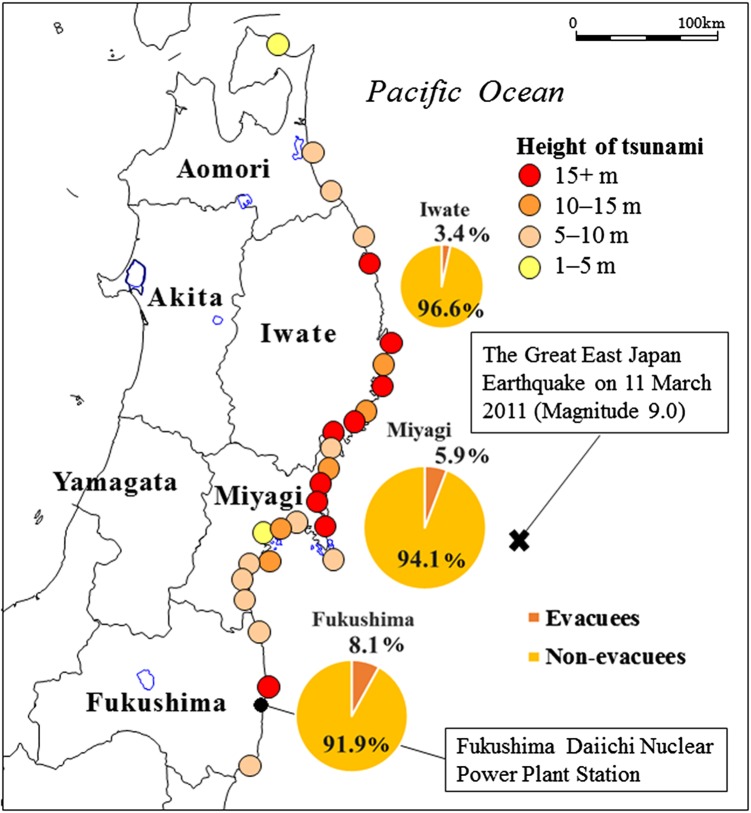
Affected and unaffected prefectures in northeast Japan.^57^ The proportions of evacuees are represented according to the numbers of evacuees in March 2012.^58^ The areas of the circles are proportional to the population size.

The great east Japan earthquake of 2011, with a magnitude of 9.0,^1^ was the fourth largest earthquake ever recorded and the largest in Japan.^2^ This earthquake, together with the subsequent tsunami^3^ and the nuclear power plant accident in Fukushima,^4^ caused immense damage to the Pacific coast of northeast Japan.^5^ The disaster resulted in a significant human and property toll: 19 466 people were killed, 6152 were injured, 124 663 houses were destroyed and 274 638 were damaged.^6^ The tragedy also affected daily life in the region, disrupting the normal eating and exercise habits of the inhabitants of Fukushima, Miyagi and Iwate prefectures (figure 1).^7^ Experts in child growth have been very concerned about the short-term and long-term detrimental health effects of the earthquake and associated events on young children.^8^ ^9^ In particular, schoolteachers and local paediatricians have focused on assessing potential weight gain among the children because the affected children mainly consumed high-carbohydrate diets after the earthquake and were not allowed to play outdoors to avoid exposure to radiation from the damaged nuclear power plant.^10^ Despite this warning about potential child obesity, there have been no reliable analyses on children's body weight since the earthquake. In addition, to the best of our knowledge, no study has investigated weight changes among resident children affected by other large natural disasters. The present study was driven by the question of whether the body mass indices (BMIs) of the children in each affected prefecture had changed relative to the BMIs of comparable but unaffected children. Furthermore, this report compared the prevalence of overweight and obese children between affected and unaffected areas in a cohort design and an ecological design.

## Methods

### Study participants and measurements

On 27 August 2012, the Ministry of Health, Labour and Welfare of Japan sent a letter to nursery schools across Japan (some of which were undestroyed and still in operation in affected prefectures) through domestic administrators requesting the recipients' participation in the Nationwide Nursery School Survey on Child Health.^11^ Nursery school records on student height and weight in the affected and unaffected prefectures of northeast Japan were collected for the participating children born between 2 April 2006 and 1 April 2007 (Japanese fiscal year 2006). Thus, the children evaluated were 4–5 years old during the month of the first primary outcome evaluation (April 2011) and were 6–7 years old when data collection was completed in 2013. Participating children were weighed in their underwear and without shoes. Measurements were based on weight scales and stadiometers, which are legally required equipment at all Japanese nursery schools and kindergartens that undergo half-yearly standardisation by certified measurers.^12^ The children were biannually assessed in April and October, and the nursery schoolteachers mailed the records on each child's height and weight to Tohoku University. Accordingly, we defined the half-yearly time points as every April and October from 2008 to 2012. The study participants were children who attended nursery schools that responded to the letter of request. Missing data included data for children who did not attend the participating nursery schools, moved out of the prefectures or died. Since there are no published data for the year 2011 of the exact number of children born in fiscal year 2006 in each prefecture, the approximate proportion of participants among the resident children was calculated according to the number of the first grade primary school students in fiscal year 2012.^13^

### Comparison of BMI changes

First, to gain an overview of the trends in children's BMIs, we represented the mean BMIs of children living in the affected prefectures facing the Pacific Ocean (ie, Fukushima, Miyagi and Iwate prefectures) and then separately the children living on the other side of northeast Japan in unaffected prefectures (ie, Yamagata, Akita and Aomori prefectures) according to a fixed-effects model that estimates chronological means.^14^ ^15^ For the representations, we separately generated four models with an explanatory variable of the time points for the three affected prefectures and the pooled unaffected prefectures. According to the estimated coefficients, we graphically plotted the mean BMIs for the affected prefectures and the unaffected prefectures. Second, for the primary comparisons of interest using a retrospective cohort design, we compared the BMI changes among the children from affected versus unaffected prefectures. Owing to the difficulty in comparing BMIs of growing children between different areas, the BMI changes after the earthquake were evaluated from a reference baseline time point of October 2010, the last measured time point prior to the date of the earthquake (11 March 2011), through April 2011, October 2011, April 2012 and October 2012. We compared the BMI changes of children in each affected prefecture with those in the unaffected prefectures using a repeated-measures analysis of variance model for mean changes from baseline^16^ (difference in BMI) for a difference-in-difference analysis of longitudinal data.^15^ A binary explanatory variable was set for whether children lived in an affected or unaffected area, and the analyses were adjusted using a covariate of age in month. The following fixed-effects model was employed:
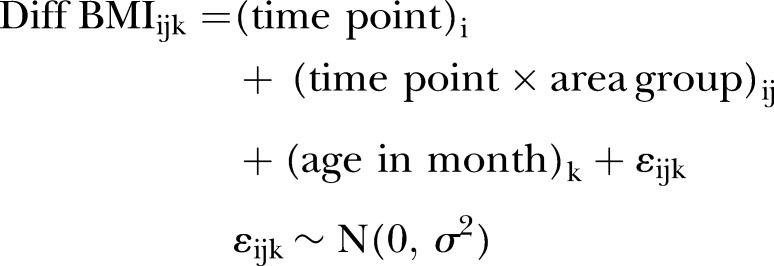
where i represents index time points of October 2010, April 2011, October 2011, April 2012 or October 2012; j represents indices for each affected prefecture or unaffected area; and k represents indices for individuals. (time point)_i_ equals zero when i equals October 2010 (the reference baseline time point). (time point×area group)_ij_ represents for an interaction term between (time point)_i_ and (area group)_j_, and equals zero at any time points when j equals unaffected area. (age in month)_k_ is a covariate of adjustment for child age. The ε_ijk_ represents the random effect of the error term in the model.

Consequently, we applied three models for comparison of BMI changes within the three affected prefectures with a single reference for the unaffected prefectures. According to the coefficients in the model, we also graphically plotted the BMI changes and then statistically evaluated the differences in BMI change between each affected prefecture and the unaffected prefectures with the statistical significance of the interaction term. All statistical analyses were performed with sex stratification using SAS statistical software (V.9.4, SAS Institute, Cary, North Carolina, USA). Descriptive statistics are reported as means and standard deviations (SDs)/standard errors (SEs). All reported p values are from two-sided analyses, with p values <0.05 considered statistically significant.

### Comparison in the prevalence of overweight and obese children

For the secondary comparisons, the prevalence of overweight and obese children was compared between the affected and unaffected prefectures from October 2010 to 2011. Overweight and obesity were diagnosed according to the child growth standards of the WHO.^17^ Since these diagnostic standards essentially change when children reach the age of 61 months, these secondary outcome comparisons were restricted to the data until October 2011 when almost half of the children were 60 months of age or younger. Although the difference in the changes in prevalence (ie, proportions) between affected and unaffected prefectures is of interest, there is no published statistical test for such difference-in-difference analysis in proportional data. Instead, we applied Fisher's exact test to evaluate the difference in the prevalence of overweight and obese children between affected and unaffected prefectures, which were stratified according to date.

In addition, for an ecological study design, the prevalence of overweight boys and girls in the affected prefectures and throughout Japan was assessed for children aged 6–17 years attending primary, junior high and high schools using the descriptive data provided through the School Health Statistics Research of Japan.^18^ The investigation by the School Health Statistics Research, which selects examined schools in a stratified random sampling under Japanese law,^19^ is conducted annually from April to June. Owing to widespread school dysfunction immediately after the disaster in March 2011, the examination could not be conducted in the three affected prefectures in 2011. Therefore, we compared the prevalence of overweight children from the 2012 examination with the prevalence from the 2010 examination for the three prefectures and across Japan to determine whether or not the prevalence had increased after the earthquake. The sources for nationwide data comprised 4.8% of all Japanese schoolchildren in 2010 and 4.9% in 2012. In 2010 and 2012, 270 720 primary schoolchildren aged 6−11 years, 225 600 junior high school students aged 12−14 years and 126 900 high school students aged 15−17 years were included. With these data, the definition of being overweight was weighing 20% or greater than a standard weight, where per cent overweight=(measured weight−standard weight)×100/standard weight for each given age, sex and height in accordance with the guidelines of The Japanese Society for Pediatric Endocrinology.^20^ ^21^

## Results

### Comparison of BMI changes

The data for the affected children on approximately 8.8% of resident children were collected from 646 boys and 597 girls who attended 97 nursery schools in Fukushima, 904 boys and 854 girls from 132 nursery schools in Miyagi and 483 boys and 458 girls from 81 nursery schools in Iwate. The data for the unaffected children on approximately 12.3% of resident children were collected from 307 boys and 285 girls attending 42 nursery schools in Yamagata, 762 boys and 739 girls from 88 nursery schools in Akita, and 638 boys and 634 girls from 108 nursery schools in Aomori.

**Figure 2:**
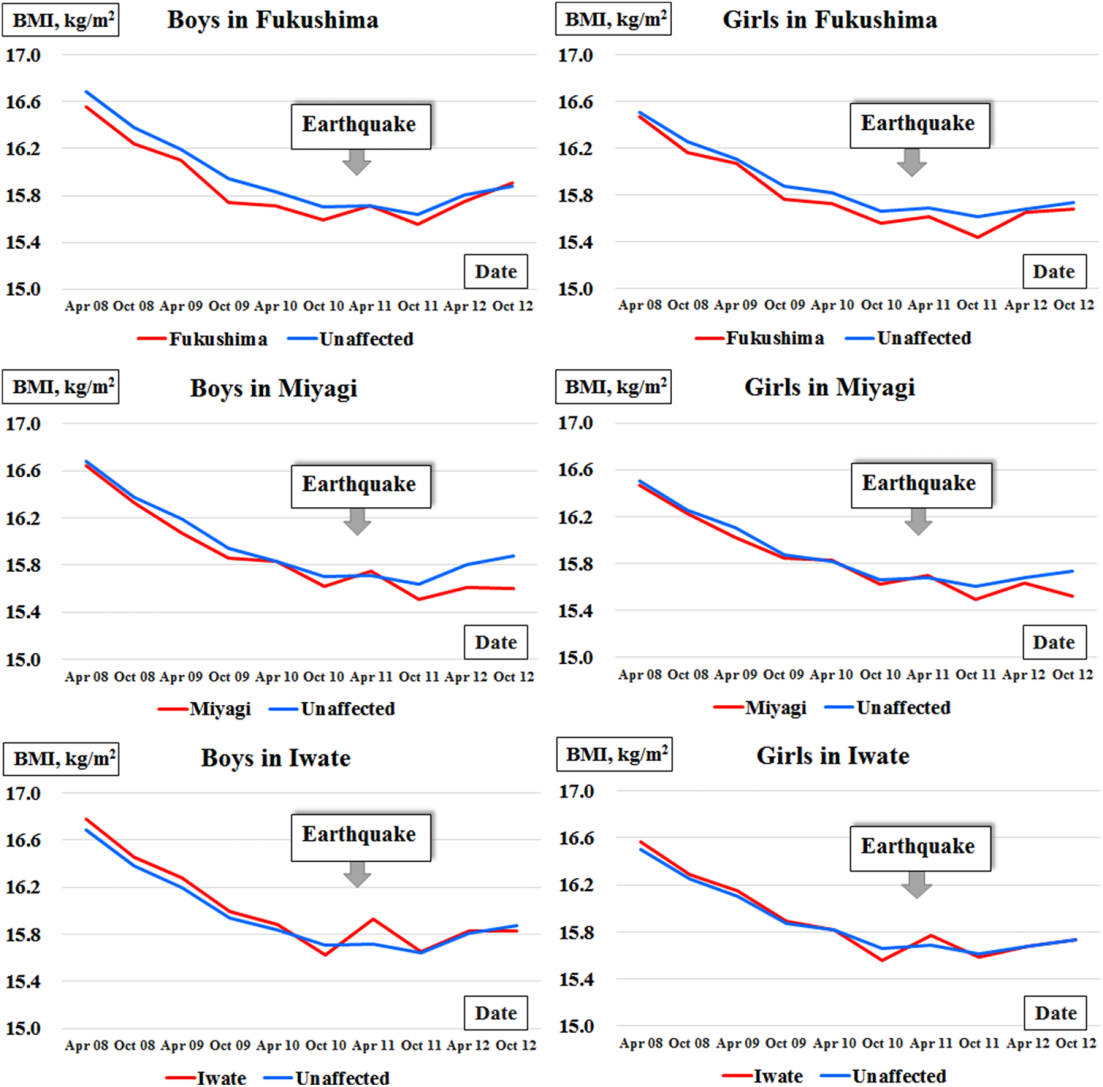
Mean body mass indices (BMIs) of nursery schoolchildren born between 2 April 2006 and 1 April 2007 in each affected prefecture versus unaffected prefectures in northeast Japan.

**Figure 3:**
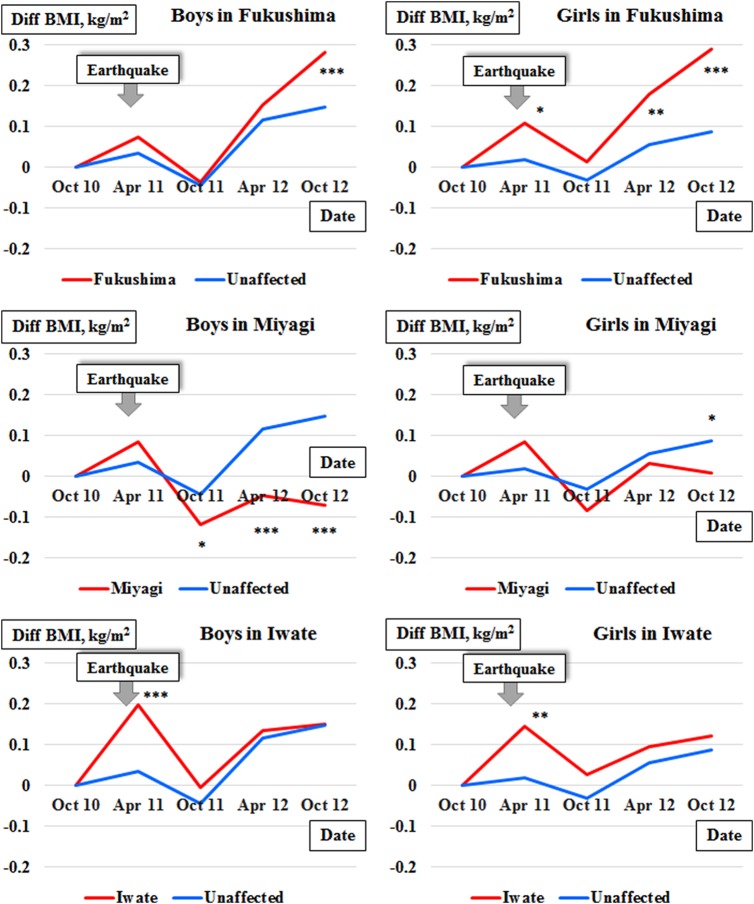
Estimated changes in body mass index (difference in BMI) after October 2010 among nursery schoolchildren born between 2 April 2006 and 1 April 2007 in each affected prefecture versus unaffected prefectures in northeast Japan. Statistical tests evaluated the p values of the interaction terms in the model. *p<0.05, **p<0.01 and ***p<0.001.

Table 1 shows the baseline anthropometrics in October 2010. Figure 2 and online supplementary table S1 present the estimated BMIs from April 2008 to October 2012 graphically and numerically, respectively. For the primary comparisons, figure 3 and table 2 illustrate the estimated changes in BMI from October 2010 to October 2012 graphically and numerically, respectively, for children residing in each affected prefecture in comparison with those residing in the unaffected prefectures.

10.1136/bmjopen-2015-010978.supp1 Supplementary data

**Table 1:** Baseline characteristics of participating boys and girls in October 2010 in northeast Japan

	Affected prefectures		Unaffected prefectures	
Anthropometric measurements	Boys (n=2033)	Girls (n=1909)	Boys (n=1707)	Girls (n=1658)
Age, years	4.1 (0.3)	4.1 (0.3)	4.1 (0.3)	4.1 (0.3)
Height, cm	100.6 (4.3)	99.6 (4.1)	100.8 (4.2)	100.0 (4.2)
Weight, kg	15.9 (2.0)	15.5 (1.9)	16.0 (1.9)	15.6 (2.0)
Body mass index, kg/m^2^	15.7 (1.2)	15.6 (1.3)	15.6 (1.2)	15.6 (1.3)

All values are presented as mean (SD).

**Table 2:** Estimated changes in mean body mass index of preschool children after the great east Japan earthquake in the affected (Fukushima, Miyagi and Iwate) prefectures and unaffected prefectures in northeast Japan

Time point	Affected prefecture	Unaffected areas*	Interaction term vs unaffected areas*	p Value for interaction term
Fukushima, boys (n=646)				
October 2010	0	0	0	–
April 2011	+0.074	+0.036	+0.040	0.29
October 2011	−0.035	−0.043	+0.011	0.78
April 2012	+0.154	+0.116	+0.041	0.28
October 2012	+0.282	+0.148	+0.137	0.0003
Fukushima, girls (n=597)				
October 2010	0	0	0	–
April 2011	+0.110	+0.021	+0.087	0.023
October 2011	+0.014	−0.030	+0.042	0.27
April 2012	+0.180	+0.056	+0.122	0.0013
October 2012	+0.290	+0.088	+0.200	<0.0001
Miyagi, boys (n=904)				
October 2010	0	0	0	–
April 2011	+0.086	+0.036	+0.048	0.14
October 2011	−0.117	−0.043	−0.076	0.018
April 2012	−0.047	+0.116	−0.165	<0.0001
October 2012	−0.069	+0.148	−0.218	<0.0001
Miyagi, girls (n=854)				
October 2010	0	0	0	–
April 2011	+0.085	+0.021	+0.061	0.057
October 2011	−0.084	−0.030	−0.057	0.077
April 2012	+0.033	+0.056	−0.026	0.42
October 2012	+0.009	+0.088	−0.082	0.011
Iwate, boys (n=483)				
October 2010	0	0	0	–
April 2011	+0.200	+0.036	+0.165	<0.0001
October 2011	−0.004	−0.043	+0.040	0.32
April 2012	+0.135	+0.116	+0.020	0.62
October 2012	+0.152	+0.148	+0.006	0.88
Iwate, girls (n=458)				
October 2010	0	0	0	–
April 2011	+0.146	+0.021	+0.124	0.0019
October 2011	+0.028	−0.030	+0.057	0.15
April 2012	+0.095	+0.056	+0.038	0.33
October 2012	+0.123	+0.088	+0.034	0.40

All values are reported as kg/m^2^.

*Unaffected refers to Yamagata, Akita and Aomori prefectures in northeast Japan.

Compared with the unaffected prefectures, the observed change in BMI in Fukushima prefecture was significantly higher among boys in October 2012 (+0.137 kg/m^2^, p=0.0003) and among girls in April 2011 (+0.087 kg/m^2^, p=0.023), April 2012 (+0.122 kg/m^2^, p=0.0013) and October 2012 (+0.200 kg/m^2^, p<0.0001).

Compared with the unaffected prefectures, the observed change in BMI in Miyagi prefecture was significantly lower among boys in October 2011 (−0.076 kg/m^2^, p=0.018), April 2012 (−0.165 kg/m^2^, p<0.0001) and October 2012 (−0.218 kg/m^2^, p<0.0001) and among girls in October 2012 (−0.082 kg/m^2^, p=0.011).

Compared with the unaffected prefectures, the observed change in BMI in Iwate prefecture was significantly higher among boys in April 2011 (+0.165 kg/m^2^, p<0.0001) and among girls in April 2011 (+0.124 kg/m^2^, p=0.0019).

### Comparison of the prevalence of overweight and obese children

**Figure 4:**
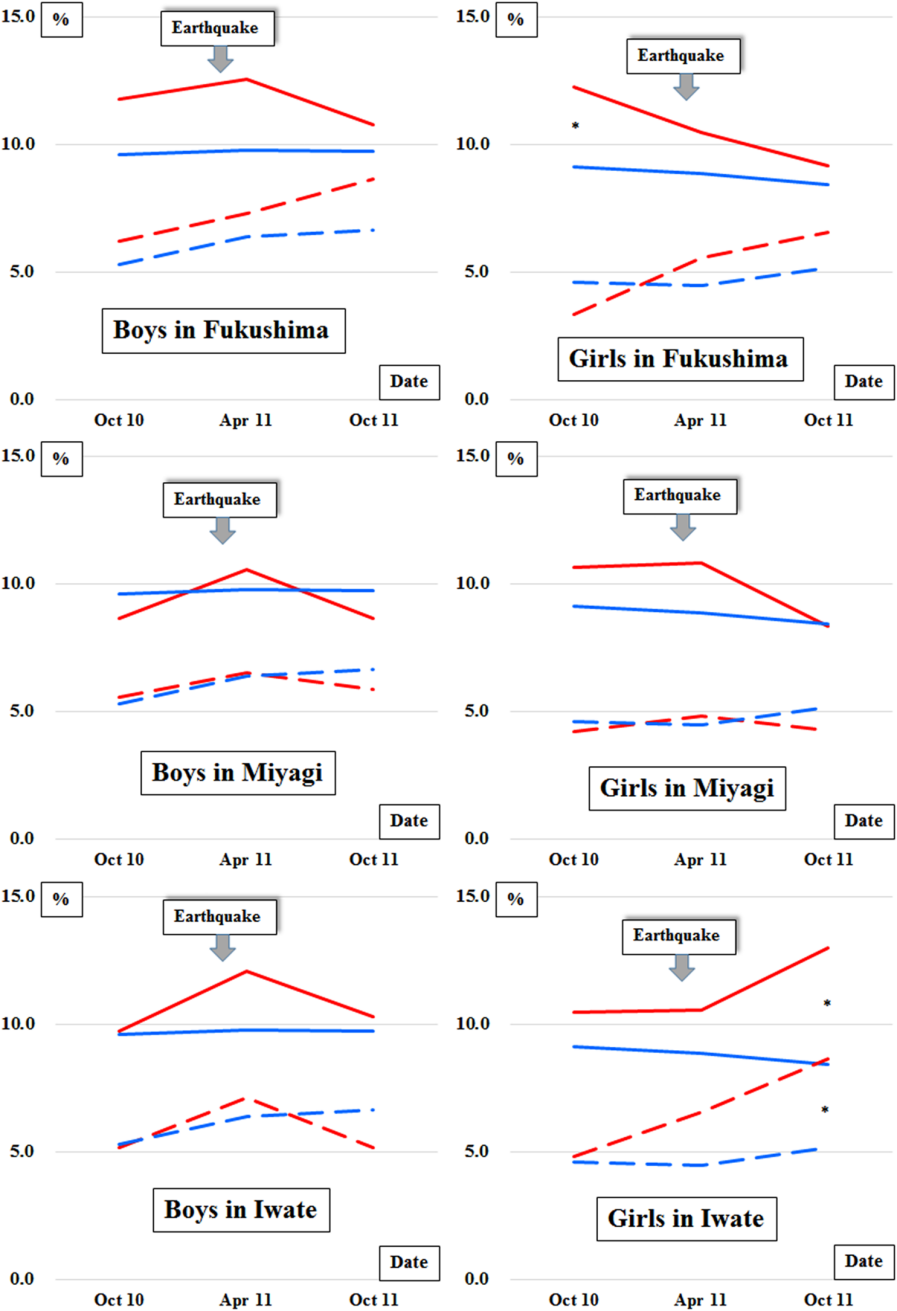
Prevalence of overweight and obese children in Fukushima, Miyagi and Iwate prefectures (red lines) and the unaffected prefectures (blue lines). Solid and dashed lines represent the prevalence of overweight and obese children, respectively. Overweight and obesity were diagnosed according to the child growth standards of the WHO.^17^ *p<0.05, **p<0.01 and ***p<0.001.

Figure 4 shows the secondary comparisons of the prevalence of overweight and obese children in Fukushima, Miyagi and Iwate prefectures with the pooled population of the unaffected prefectures. Compared with the unaffected prefectures, there was a slight increase in the changes of the prevalence of overweight boys between October 2010 and April 2011 residing in Fukushima, Miyagi and Iwate. In contrast, there was a slight decrease in the change of the prevalence of overweight girls residing in Fukushima. Compared with the unaffected prefectures, a slight increase in the changes of the prevalence of obese individuals between October 2010 and April 2011 was observed among boys in Iwate and among girls in Fukushima, Miyagi and Iwate. In contrast, a slight decrease in the change of the prevalence of obese boys was observed in Miyagi. The ecological study also compared the prevalence of overweight children in the affected prefectures as well as across Japan in 2010 and 2012 (see online supplementary figure 1). We observed increases in the prevalence of overweight individuals among primary schoolboys in the 6−11 age group in Fukushima, the 6−12 age group in Miyagi, the 6−9 age group in Iwate and the 6−10 age group across Japan. We also found increases in the prevalence of overweight primary schoolgirls in the 6−10 age group in Fukushima, the 8−11 age group in Miyagi and the 6−11 age group across Japan. Among the girls aged 6−7 years in Miyagi, we also observed a slightly decreased prevalence of overweight children. No noteworthy change in the prevalence of overweight children was observed among primary schoolgirls (6−12 years) in Iwate. In the three affected prefectures and across Japan, there were no consistent trends in the prevalence of overweight individuals among junior high and high school students aged 12−17 years.

## Discussion

### Main results

Our data on postdisaster BMI changes (figure 3) showed immediate increases in BMI among the preschool boys and girls residing in each affected prefecture, as if in response to the disaster in March 2011. In addition, there was evidence of a prolonged increase in BMI among the boys and girls residing in Fukushima. On the other hand, in Miyagi, we identified a trend of immediate weight gain with subsequent weight loss in boys and girls. In Iwate, the BMIs of boys and girls gradually approached those of the children living in unaffected prefectures. The prevalence of obese individuals in the cohort data increased 1 month after the earthquake among boys in Iwate and among girls in the three affected prefectures, compared with that in the unaffected prefectures (figure 4). In the ecological study (see online supplementary figure 1), there were increases from 2010 to 2012 in the prevalence of overweight boys and girls in Fukushima and overweight boys in Miyagi and Iwate in their early primary school years, although the results were inconsistent among girls in Miyagi and Iwate who were also in early primary school years. Although the psychological harm that natural disasters cause to children has been reported,^22^ the present results have provided additional evidence of an immediate and potentially prolonged increase in BMI among young children following a major disaster.

### Possible explanations

At the time of the 2011 earthquake, electricity, gas lines, water supply lines, sewage systems, railways and traffic transportation were all interrupted.^5^ The interruption in daily transportation resulted in severe shortages of meat, fish, egg and vegetables.^23^ As typically occurs with disasters, administrative and non-political/non-profit organisations supplied carbohydrates such as rice balls and bread to affected populations.^8^ ^24^ In the three affected prefectures, the priority was to supply meals to the affected children.^25^ The affected children are presumed to have gained weight due to the carbohydrate-based diet that was supplied, which may account for the weight gain observed in boys and girls in the three affected prefectures immediately after the earthquake, either with or without statistical significance (figure 3). Furthermore, school gymnasiums were used as shelters for evacuees, and school playgrounds were opened for provisional housing at that time.^24^ In Fukushima, where the nuclear power plant station experienced hydrogen explosions, there were few chances to play outside due to fear of radiation exposure, the lack of available playgrounds and an overall mournful mood among the population. Additionally, on 19 April 2011, the Ministry of Education, Culture, Sports, Science and Technology of Japan issued a notice to all schools in Fukushima that principals needed to restrict the availability of school buildings and playgrounds as long as the schools were exposed to 1 mSv or more of radiation per year.^26^ The limited outdoor activity may have been reflected in the prolonged BMI increases observed among children living in Fukushima. In contrast, in Miyagi, city infrastructure, hospitals, school education and corporate activities have been recovering much sooner than in Fukushima.^27^ ^28^ This contrast may in part explain the different trends in weight loss observed among boys and girls living in Miyagi. However, how they lost weight has not been established. In Iwate, one report has described a worsening of the mean plasma glucose and glycated haemoglobin levels in 63 affected patients with diabetes from 109.4 mg/dL (SE, 3.9, 6.08 mmol/L (SE 0.22)) to 134.3 mg/dL (SE, 7.2, 7.46 mmol/L (SE 0.40)) and from 5.9% (SE, 0.2, 6.8 mmol/L (SE 0.3)) to 6.5% (SE, 0.2, 7.8 mmol/L (SE 0.3)), respectively, 4 months after the earthquake.^29^ The authors were physicians in charge of following up with these patients and they witnessed unbalanced diets heavy with sweets, canned products and boil-in-the-bag foods provided to the evacuees. The authors have speculated that the worsening of the glycaemic control was partly due to unbalanced diets. As was the case in Fukushima and Miyagi, the reported situation of limited access to an adequate diet in Iwate may partly explain the immediate BMI increases observed among preschool boys and girls after the earthquake. Since the Iwate prefecture is relatively far from the epicentre of the earthquake and the damaged nuclear power plant (figure 1), the daily lives of its inhabitants may have returned to normal sooner than it did for those in Fukushima.^27^ ^28^

### Comparison with previous studies

Extraordinary experiences during major disasters change the lives of inhabitants and lead to an array of physical and mental problems.^30–32^ A study from medical examinations has shown a +0.2 to +0.3 kg/m^2^ BMI change in a year among Fukushima evacuees.^33^ Another report from a cohort in Miyagi has described a +0.25 kg/m^2^ BMI change among city officials engaging in postquake recovery and a −0.09 kg/m^2^ BMI change among the general population in 2011 after adjusting for sex and age.^34^ Our results comparing children in Fukushima and Miyagi are consistent with these previous reports that investigated changes among adults. As described above, the observed BMI increases immediately after the disaster may be partly attributed to the unbalanced diet and elevated hormone levels induced by psychological stresses. At the 1995 Great Hanshin (Kobe) earthquake in Japan (7.3 magnitude on the Richter scale), physicians have also reported worsened glycaemic controls among patients with diabetes.^35^ The authors, who were members of disaster relief teams, have explained that the exacerbations were partly due to unhealthy high-carbohydrate diets and overeating, in responses to sleeplessness and a fear of hunger. At the 2004 Mid-Niigata earthquake (6.8 magnitude on the Richter scale), a report from health check-up data for overworked male prefectural governmental staff members has described an average+0.2 kg/m^2^ yearly BMI increase among victims and an average+0.1 kg/m^2^ yearly BMI increase among non-victims.^36^ At the 1999 Taiwan earthquake (7.2 magnitude on the Richter scale), a study reported that increased sympathetic hormone levels of leptin and cortisol are associated with hyperarousal.^37^ Since leptin regulates food intake and body weight,^38^ and cortisol is induced by psychological stress,^39^ hormonal changes in hypervigilant individuals following major earthquakes may cause disturbances in their appetite and BMI.

### Practical implications

Medical attention pertaining to mental health and lifestyle-related issues must be provided to adults and, in particular, elderly adults with a chronic condition during earthquake recovery.^40^ With most natural disasters, the focus has been traditionally placed mainly on the health problems of adults and not on the needs of children. This study sheds new light on the risks that a disaster can pose to childhood growth and their risk of obesity after a disaster. The mean BMI levels among boys in Fukushima and boys and girls in Iwate, all approximately 4 years old at the time that the earthquake struck, appear to show a relatively earlier adiposity rebound with immediate and prolonged weight gain (figure 2). In paediatrics, adiposity rebound is defined as the point of the minimal BMI that comes at 5–6 years of age on average.^41^ There is a consensus that early adiposity rebound predicts diabetes and obesity in adulthood,^42^ ^43^ although discussion continues about whether the reason for undesirable outcomes at adult age is due to children's lifestyles,^44^ to their fetal lives^45^ or to other causal mechanisms.^46^ Hence, if earlier adiposity rebound indeed occurs in a subset of children after natural disasters due to lack of diet and exercise, administrative agencies and local paediatricians should pre-emptively address this source of future cardiovascular diseases. Since being physically active during the preschool ages reduces BMI over a long term,^47^ in the immediate aftermath of an earthquake, play space availability should be ensured, balanced diets should be supplied and schools should be reopened at the earliest possible date. Additionally, endocrinological and metabolic abnormalities often appear in preschool children with a 12-month history of being overweight.^48^ Indeed, stress experienced in early childhood can persist and cause future neurological and endocrine-related cardiovascular disease.^49^ Thus, paediatricians need to ensure long-term follow-up and pay close attention to the health of children affected by a disaster.

### Limitations and strengths

This study had several limitations. The primary limitation was the representativeness of the sample populations in affected prefectures of northeast Japan. The registered children with available data attended nursery schools that responded to the letter of request. Therefore, the data did not include children who died; those in destroyed nursery schools, nursery schools without schoolteachers or other deficiencies or those who had moved away from the area. Since data were not available indicating whether the most severely affected children gained or lost weight, the direction and the amount of this bias in BMI were not determined. Conversely, the study design could have specifically focused on children who experienced severe suffering. Owing to the study design, the definition of ‘affected’ children did not identify those who were evacuated to provisional houses or who were physically impacted by the tsunami. Therefore, the observed influence of the disaster on their child growth may have been diminished, and the data may not reflect all children in the affected prefectures. However, if the bias should exist, the effects of the earthquake on BMIs would be attenuated according to the observed data and bias towards the null hypothesis. Thus, we consider that the conclusions from the attenuated results would be held. Additionally, since nursery schools in Japan require that either both parents or a single parent without a spouse should be employed, nursery school students may not represent the socioeconomic status of all children in the studied prefectures. Therefore, although the comparisons of nursery school students in northeast Japan can be internally valid, it may not be possible to generalise the results to all preschool children who will be affected by another disaster. Second, the lack of information on diet and physical activity may limit the comparability of outcomes between the affected and unaffected prefectures studied. Since the Pacific Ocean side of northeast Japan receives less snow than the opposite side, exercise may be more frequent in the affected prefectures than in the unaffected prefectures. This cultural factor may induce bias towards decreasing BMIs of the affected children residing on the Pacific Ocean side. Considering this negative bias in BMI, the weight gains among children living in Fukushima and Iwate might be larger, and the weight loss observed in Miyagi might be smaller than thought. Since there are no published data for the difference in BMI between growing children residing on the Pacific Ocean side or the other side of northeast Japan, the amount of this potential bias was undetermined. To correct for this potential bias, study initiation with a matching method based on cultural confounders for a quasi-experimental design might have reduced the bias. Even so, we minimised the bias by selecting an unaffected reference group from northeast Japan, where the diet was considered to be similar to that in the three affected prefectures.^50^ Finally, the results were limited with no use of z score (SD score) for BMI,^51–53^ which might have more properly adjusted for age. Although the standardisation by z score may be ideal for comparison of raw BMI values, the need for comparison of the BMI changes did not allow us to use the standardisation. Thus, we instead chose to compare BMI changes between two groups and make the simple adjustment of a covariate for months of age in the model.

The assembled longitudinal data would be strengthened by its uniqueness in recording child growth before and after a disaster. Although medical attention to the physical and mental health of people affected by a disaster has recently increased, surveys pertaining to this particular disaster have just begun.^54^ A number of studies originating from these surveys should provide evidence to bolster disaster medicine. Another strength of this study is comparison of affected children with unaffected children, who were considered to have been growing normally. For example, although one report described the health status of Iraqi refugees before immigration to the USA with an obesity prevalence of 24.6% and a hypertension prevalence of 15.2%,^55^ the lack of information on unaffected Iraqis prevented an estimation of the influence of refugee life on human health. Similarly, the impact of a study that reported a high prevalence of mental disorders in Iraqi children during a war^56^ would be weakened because of the lack of an unexposed comparator reference group. The evaluation of BMIs in growing children is usually difficult. However, we believe that an epidemiological answer has been provided to the study question on whether children's BMIs were influenced by the disaster. Furthermore, the phenomenon of an increased prevalence of overweight early-year primary schoolchildren in Fukushima has been observed in the ecological study. Although an ecological fallacy may exist, it is interesting that this phenomenon has appeared in Fukushima, where there are reports of delayed reconstruction.

## Conclusion

The data from earthquake-stricken northeast Japan have shown an immediate increase in BMI among children living in three affected prefectures. The data have also indicated trends of prolonged BMI increases among children in Fukushima and prolonged BMI decreases among children in Miyagi. These data emphasise the need for attention to and follow-up for affected children after a natural disaster to prevent undesirable health outcomes.
