# Supplementary material for: Impact of the great east Japan earthquake on the body mass index of preschool children: a nationwide nursery school survey
Source: BMJ Open. 2016 Apr 7;6(4):e010978. doi: 10.1136/bmjopen-2015-010978 (PMC4838714; doi:10.1136/bmjopen-2015-010978)

Supplementary Materials

Supplementary Table 1 Estimated mean body mass indices for children residing in the affected Fukushima, Miyagi and Iwate prefectures and in unaffected areas also located in northeast Japan

Time point	Boys				Girls			
	Fukushima (n = 646)	Miyagi (n = 904)	Iwate (n = 483)	Unaffected* (n = 1707)	Fukushima (n = 597)	Miyagi (n = 854)	Iwate (n = 458)	Unaffected* (n = 1658)
April 2008	16.34	16.45	16.43	16.38	16.21	16.33	16.26	16.38
October 2008	16.08	16.17	16.23	16.14	15.97	16.12	16.11	16.14
April 2009	15.97	15.99	16.05	16.00	15.96	15.90	15.95	16.00
October 2009	15.73	15.83	15.85	15.83	15.78	15.79	15.79	15.83
April 2010	15.73	15.81	15.76	15.78	15.73	15.76	15.73	15.78
October 2010	15.63	15.65	15.57	15.64	15.66	15.62	15.56	15.64
April 2011	15.73	15.74	15.75	15.64	15.74	15.70	15.69	15.64
October 2011	15.63	15.54	15.59	15.56	15.61	15.52	15.58	15.56
April 2012	15.78	15.61	15.70	15.71	15.82	15.61	15.61	15.71
October 2012	15.88	15.62	15.74	15.73	15.91	15.59	15.64	15.73

All values are reported as kg/m^2.

*Unaffected refers to three unaffected prefectures of northeast Japan (Yamagata, Akita and Aomori).

Supplementary Figure 1 Prevalence of overweight children in Fukushima, Miyagi and Iwate prefectures and throughout Japan in 2010 versus 2012 from the School Health Statistics Research of Japan.[18] The term 'overweight' was defined as weighing 20% or more than standard weight in accordance with the guidelines of The Japanese Society for Pediatric Endocrinology.

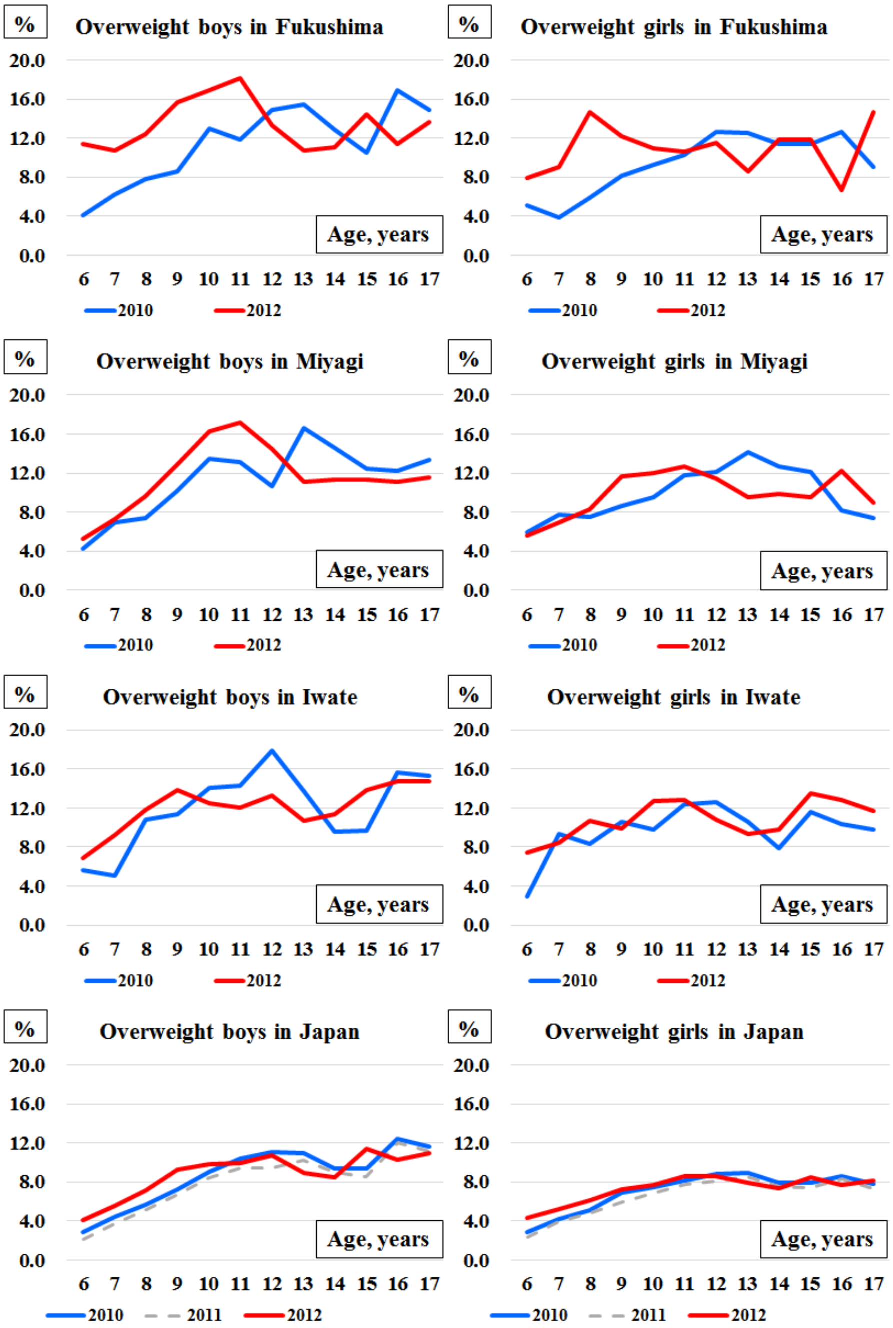

Supplement: Supplementary data [file bmjopen-2015-010978supp.pdf]
